# Use of peers, community lay persons and Village Health Team (VHT) members improves six-week postnatal clinic (PNC) follow-up and Early Infant HIV Diagnosis (EID) in urban and rural health units in Uganda: A one-year implementation study

**DOI:** 10.1186/s12913-015-1213-5

**Published:** 2015-12-15

**Authors:** Zikulah Namukwaya, Linda Barlow-Mosha, Peter Mudiope, Adeodata Kekitiinwa, Joyce Namale Matovu, Ezra Musingye, Jane Ntongo Ssebaggala, Teopista Nakyanzi, Jubilee John Abwooli, Dorothy Mirembe, Juliane Etima, Edward Bitarakwate, Mary Glenn Fowler, Philippa Martha Musoke, Peer-senga study Group at Mulago, Mengo, Rubaga and Mpigi Health Units

**Affiliations:** Makerere University-Johns Hopkins University (MU-JHU) Research Collaboration, Upper Mulago Hill Road, P.O. BOX 23491, Kampala, Uganda; Baylor College of Medicine Children’s Foundation, Kampala, Uganda; Makerere University College of Health Sciences, Pediatrics and Child Health, Kampala, Uganda; Mpigi Health Centre IV, Mpigi, Uganda; Elizabeth Glaser Pediatric AIDS Foundation, Kampala, Uganda

**Keywords:** PMTCT, HIV, Postnatal, Peers mothers, Community lay persons, Early Infant Diagnosis

## Abstract

**Background:**

Effective Prevention of Mother to child Transmission of HIV (PMTCT) relies heavily on follow-up of HIV-infected women and infants from antenatal, through postnatal, to the end of the breastfeeding period. In Uganda, postnatal (PNC) follow-up remains below 50 % creating a missed opportunity for linkage to comprehensive HIV care and early infant diagnosis (EID). We evaluated the use of HIV infected peer mothers (peers), community lay persons and Village health team (VHT) members to improve PNC follow up and EID in urban and rural health units.

**Methods:**

Study participants were HIV-infected women recruited from antenatal clinics at three urban clinics (Mulago, Rubaga and Mengo hospitals) and one rural health centre (Mpigi Health centre IV) between January and September 2010. The women were followed through delivery and the mother-infant pairs for the 6-week postnatal visit and up to 14 weeks for EID. Peers, community lay persons and VHT members were identified and trained in basic PMTCT and reproductive health (RH). They were then assigned to study clinic to support and follow study participants, their partners and infants through provision of health education, counseling, home visits, and phone call reminders. Six week PNC attendance was measured as a proportion of mother-infant pairs that returned for the 6-week postnatal follow up visit (5–8 weeks) while EID was measured as the proportion of HIV-exposed live birth that had an HIV test done by 14 weeks of age. Data at baseline (one year before the intervention) was compared with that during the one year study period among study participants and HIV infected women and their HIV-exposed infants in the whole clinic population.

**Results:**

A total of 558 HIV-infected pregnant women were recruited for the study, 47 mother-infant pairs were censured before 6 weeks due to stillbirth (14), infant death < 6 weeks (23), death of participant (04) and loss to follow up before delivery (6). 401/511 (78.5 %) of mother-infant pairs returned to the study clinics at six-week, while 441/511 (86.3 %) infants were tested for HIV infection by 14 weeks of age. The baseline six-week PNC follow up was 37.7 % and increased during the study period to 78.5 % and 39.1 % among study participants and whole clinic population respectively, an incremental difference of 39.4 % (*P* < 0.001). EID increased from a baseline of 53.6 % to 86.3 % and 65.8 % among study and whole clinic population respectively during the study period, an incremental difference of 20.5 % (*P* < 0.001).

**Conclusions:**

Use of peers, community lay persons and VHT members led to a significant increase in six-week postnatal follow up of HIV infected women and EID among HIV exposed infants in the four study clinics. Our study supports the use of peers to improve early postnatal follow up and EID and should be implemented in other health units to support the PMTCT cascade.

## Background

The World Health Organization (WHO) recommends that every pregnant woman should attend a minimum of four antenatal (ANC) visits, deliver with the assistance of a skilled provider, receive a full postnatal care (PNC) package following delivery and the baby to receive appropriate newborn and child care [1–3]. However, from 2005 to 2012, only 37 % of pregnant women in low income countries attended all four required ANC visits, 47 % were delivered by a skilled birth attendant and postnatal attendance was only about 30 % [4–6].

WHO further recommends that HIV exposed infants should receive HIV testing at the earliest opportunity, from six-weeks of age, and ART initiated for all infected infants below 5 years of age [7]. The six-weeks PNC visits is particularly important in the PMTCT follow-up cascade as it is the access point for Early infant HIV diagnosis (EID), growth monitoring and evaluation, cotrimoxazole prophylaxis initiation and infant feeding counseling and support. [8, 9] Subsequent postpartum visits for the mother and baby are important to communicate infant HIV results to the caregiver, support safe infant feeding options and growth monitoring [10, 11].

In Uganda, low postnatal follow up of infants remains a major contributor to the low EID rates, with only 40.2 % of HIV exposed infants receiving an HIV test. [12] Furthermore, only 40 % of the infants identified as HIV-positive through the EID program are linked to HIV care and treatment programs. [9, 13] Major factors identified for these low follow up rates include gaps in identifying and referring infants to EID care points, failure to return results to the caregiver and failure to link the HIV-positive infants to ART care [7, 14, 15].

Improving linkages and creating a continuum of care in PMTCT is critical in ensuring optimal service delivery. The “Tingathe-PMTCT” pilot intervention in Malawi used Community Health workers to achieve over 80 % EID among 1318 HIV exposed infants born in two peri-urban communities in Malawi [16]. This level of task shifting (delegation of medical tasks, where appropriate, to less specialized health workers, including trained lay persons) has expanded the human resource pool, including bridging the health facilities to the community [17–19]. In South Africa, the mother2mother project has successfully employed and trained mothers living with HIV to work alongside doctors and nurses, offering support and education to their peers [20, 21].

In this study, we trained peers, influential community lay persons (“*sengas” &”kojjas”*) and Village Health Team (VHT) members to equip them with skills in community mobilization and educational key messages for HIV/AIDS and reproductive health activities with the aim of increasing the proportion of mother-infant pairs returning for their six-week postnatal follow-up and the infant receiving EID for HIV infection by 14 weeks of age to at least 70 % in three urban clinic sites in Kampala and one rural health center IV in Mpigi.

## Methods

This community-based operational research study was conducted at three urban hospitals including Mulago national referral hospital (urban-free medical services), Rubaga and Mengo hospitals (urban-private not-for-profit general hospitals) located within Kampala city and one rural health centre IV (rural-free medical services) in Mpigi district in Uganda. Mulago hospital conducts over 33,000 deliveries annually with an HIV sero-prevalence of about 10 %. Rubaga and Mengo Hospitals each conducts about 6000 deliveries annually with an HIV sero-prevalence of 7 %. These three urban hospitals have a catchment population of approximately two million people, coming from Kampala and the surrounding districts. Mpigi health center is located 40 km west of Kampala and registers over 4000 pregnant women in ANC and 2000 deliveries annually. The HIV sero-prevalence at Mpigi health center IV is 5.5 %. The HIV/PMTCT services at all the study sites were offered free of charge to all client who sought ANC, delivery and postnatal reproductive health services, even at the private not-for-profit hospitals. Women identified to be HIV infected received zidovudine (AZT) from 28 weeks of gestation, followed by single doze nevirapine (sdNVP) at the onset of labour then AZT and Lamivudine (3TC) for one week as a tail to reduce NVP resistance, in line with the Uganda national PMTCT guidelines. Infants received sdNVP syrup at birth.

### Peers, community lay persons and VHT members

The women in the study received additional counseling, home visiting and community sensitization by peers, community lay men (“*kojjas”*) community lay women (“*sengas”*) and village health team (VHT) members. Members of the VHT were selected from the existing district VHTs in rural Mpigi, and further trained to support and follow up study participants. The recruitment of peers, community lay persons and VHT members involved consultations with local leaders and community based organizations (CBOs). The recruitment criteria for peers, community lay persons and VHT members included: 1) ability to read and write, 2) permanent residence in the respective community, 3) respected in the community, 4) experience in community health mobilization, and 5) willingness to volunteer during the study period. In addition to the above criteria, peers were selected who were HIV infected, had to have previously worked or volunteered at an HIV clinic, and were willing to be participant confidant. Peers had disclosed their HIV status to immediate family members and were willing to share care and treatment testimonies with the study participants.

In total, 95 lay persons in Kampala (urban) and 10 VHT members in Mpigi (rural) were selected and trained to give key ANC messages and follow up of the women and their babies. The number of peers recruited at the sites included: Mulago (4), Mengo (2), Rubaga(2) and Mpigi(2). A five-day training workshop was held with the peers, lay persons and VHT members to equip them with skills in community mobilization and educational key messages for HIV/AIDS and reproductive health activities, including participant support and follow up, activity reporting and study protocol activities. The training materials used were adopted from the Uganda Ministry of Health (MoH) guidelines on community mobilization and engagement [22]. The training sessions comprised of mini lectures, group discussions, practical skills demonstrations and case scenarios. The training emphasized how to deal with stigma, discrimination and confidentiality. At the end of the baseline training each trainee developed a work plan that was followed during the study period. Follow up monthly meetings by the study team (study coordinator, research assistant and health visitor) and the community lay persons were held in the community, where each lay person submitted monthly activity report to the study team. Regular and targeted trainings were organized by the study team to update and orientate the lay persons on the study procedures. To facilitate movement in the community, VHT members were each given a bicycle with protective wear, the community lay persons were facilitated with transport allowance for the days worked (as per their monthly activity reports) and the peers received a monthly stipend (USD50-150 per month) to cover daily transport and lunch.

At screening for study enrolment, study participants were asked their preference between peers, lay persons or VHT members to home visit them and some participants declined to be followed by the community lay women or VHT members. Therefore, more home visits by peers were arranged by the study team, leaving the lay persons and VHT members to concentrate on educating and sensitizing the communities.

### Activities

The community lay persons conducted mobilization and education sessions to target mostly women of reproductive age and their partners. Opportunities to sensitize the community were ceased at any gathering including areas of worship and village meetings, but also at sports galas, burial ceremonies and open market days. The key messages included: benefits of attending ANC including PMTCT, importance of couple counseling and testing, family planning, and role of men in reproductive health. Community lay men (“*Kojjas”)* supported the male partners of pregnant women and encouraged them to be tested for HIV, to attend ANC and PNC, and follow up HIV care and treatment along with their female partners. In addition, lay persons held community meetings in the evenings and weekends to accommodate men’s work schedule. The study team attended a selected number of community meetings to support and supervise the lay persons and all these activities were summarized in the monthly activity reports.

Peers were stationed at the study health units and participated in study participant recruitment, follow up (including home visiting and phone calling), and linkage to other services within the health unit. They worked closely with the health workers and study team at the clinic to ensure that participants received the full health care package and were linked to the community lay persons. Peers also reminded participants of subsequent study and follow-up clinic appointments. They also conducted general health education sessions on couple counseling and HIV testing, infant feeding counseling, one-on-one psychosocial support with the participants and the importance of postnatal follow-up. Other study related activities by peers included: issuing invitation letters for male partners, telephone reminders of next appointment date, scheduled and unscheduled home visits for HIV infected pregnant women and their infants.

General community activities by the community lay persons, VHT members and study team included mobilization and sensitization meetings, dance and drama, mass media campaigns through popular radio and TV stations, targeted DVDs shown in local cinema halls, distribution of information, education and communication (IEC) materials, condoms and insecticide treated mosquito nets (ITN).

### Data collection, follow up and visit schedule

Study participants were HIV-infected women recruited from antenatal clinics at the four study sites. Following eligibility screening and written informed consent, a structured questionnaire was administered to collect baseline demographic, socio-economic and clinic attendance information.

The participants were followed up to delivery, during which time they consented for further follow-up with their live babies. The follow up schedule and activities for the mother-infant pairs was aligned to the PNC routine follow up visits. From six weeks of age, the babies were immunized and also blood was drawn for early infant HIV diagnosis. Peers phone-called mothers to encourage them to attend their post natal visits and also discussed the mother and baby health status. Three scheduled home visits were planned per participant: one at enrolment in the study, one during pregnancy (from 28 weeks of gestation) and another two weeks post-delivery to check on mother and the newborn. Participants were visited according to the home visit schedule or to encourage them to attend the clinic after missing a scheduled clinic or at the end of the study to complete study activities. On average, 3.5 peer home visits were made per participant.

Aggregate baseline clinic attendance and PMTCT service delivery information (July 2009-June 2010) was collected from clinic registers at each of the study sites by trained research assistants and compared with similar information one year later (July 2010-June 2011). Six weeks PNC attendance was measured as a proportion of mother-infant pairs (HIV positive women and their HIV-exposed infants) that returned for the 6 week postnatal follow up visit (5–8 weeks) while EID was measured as the proportion of HIV-exposed infants that had an HIV test done by 14 weeks of age. The whole clinic population data for HIV positive women and exposed infants presented included study participants’ data as it was not possible to disaggregate study participants’ data from the rest of the whole clinic data for this analysis. Therefore the proportion from the rest of the clinic includes data for study participants.

### Statistical methods and sample size

We recruited a total of 558 HIV infected pregnant women distributed proportionately based on the health unit antenatal clinic attendance. This sample size would give us a power of 80 % to detect a significant percentage increase of 20 %, given a two sided 5 % level of significance. We compared the percentage increase in six weeks attendance and EID for infants in the study as the difference between baseline and study implementation data, and compared it to the percentage increase for the whole HIV positive women and HIV-exposed infants’ clinic population at each site. For 6-weeks PNC visit and EID we considered women who registered live birth, minus infant or maternal death prior to 6 weeks (*N* = 511). We used the two proportion Z-test to compare PNC attendance and EID between the study participants and HIV infected women and their HIV-exposed infants in the whole clinic population at the health unit site. Logistic regression was used to examine possible factors associated with PNC attendance among the study participants.

### Ethical considerations

The study was approved by the Makerere University School of Health Sciences Ethics Committee and the Uganda National Council of Science and Technology. Permission to conduct the study was sought from the hospital administration at each of the study sites. Mothers provided written informed consent to participate in the study at enrollment during pregnancy and at birth for follow up of both mother and baby.

## Results

**Fig. 1:**
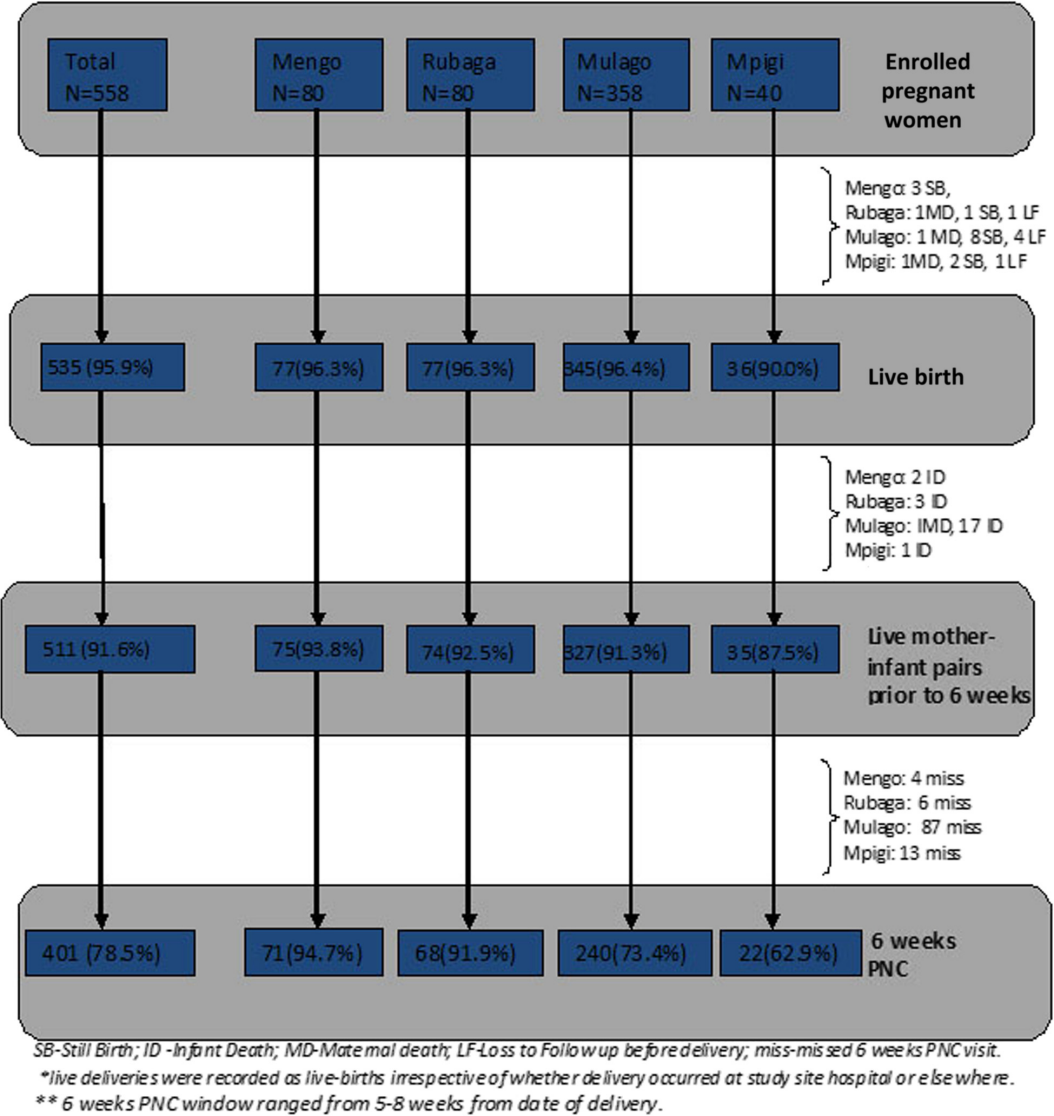
Enrollments, Live deliveries and 6 weeks PNC attendance among study participants

A total of 558 women (Mengo = 80: Rubaga = 80: Mulago = 358: Mpigi = 40) were enrolled from the four study sites between January and September 2010. Over 95 % (535/558) of the participants had a confirmed live delivery, with Mengo recording no loss to follow up before delivery. A total of 47 mother-infant pairs were censured before 6 weeks due to stillbirth (14), infant death before 6 weeks (23), death of mother participant (04) and loss to follow up before delivery (6). At six-week PNC, 401/511 infants were brought back with Mengo and Rubaga hospitals recording the highest attendance of 94.7 % (71/75) and 91.9 % (68/74) respectively, followed by Mulago at 73.4 % (240/327) and Mpigi with the lowest attendance of 62.9 % (22/35). (Fig. 1).

Loss to follow up or missed visit after delivery accounted for 21.5 % (110/511) of the mother-infant pairs that did not attend the 6-week PNC visit. Among these, some participants declined further follow up either because they were attending other clinics near their homes (15; 2.9 %) or declined further study activities all together (3; 0.6 %). Participant attendance status during the study was confirmed through clinic visits, home visit or phone contact by peers. (Fig. 1).

Women from rural Mpigi were slightly younger (median 25 years: IQR10) compared to urban-private not-for-profit (26 years: IQR 6–8) and urban-free (27 years: IQR 7) health units. Majority of participants were married/cohabiting (79.0 %; 441/558), and women from the rural Mpigi health unit were less educated (27.5 %; 11/40) and less likely to be employed (37.5 %; 15/40) compared to those from the urban hospitals (Table 1). The majority of married/co-habiting women reported their partners were employed (94.1 %; 415/441). Most women reported having disclosed their HIV status (376/558: 67.4 %) to a person of their choice; however only 44 % (194/441) of the married/co-habiting women had disclosed to their partners. The majority (81.1 %; 420/518) of women in the urban units were attending an HIV clinic at the time of enrolment compared to only 37.5 % (15/40) of women in rural Mpigi. (Table 1).

**Table 1:** Demographics and Baseline characteristics of women enrolled in the study

		Urban private		Urban free	Rural free
	Total *N* = 558 *n* (%)	Mengo *N* = 80 *n* (%)	Rubaga *N* = 80 *n* (%)	Mulago *N* = 358 *n* (%)	Mpigi *N* = 40 *n* (%)
Age in yrs, median (IQR)	26 (7)	26 (8)	26 (6)	27 (7)	25 (10)
Marital status					
Married/cohabiting	441 (79.0)	69 (86.3)	65 (81.3)	278 (77.6)	29 (72.5)
Single/separated/widowed	117 (21.0)	11 (13.7)	15 (18.7)	80 (22.4)	11 (27.5)
Education					
None/Primary	280 (50.2)	18 (22.5)	32 (40.0)	201 (56.1)	29 (72.5)
Secondary/Post-secondary	278 (49.8)	62 (77.5)	48 (60.0)	157 (43.9)	11 (27.5)
Employed					
Yes	254 (45.5)	44 (55.0)	40 (50.0)	155 (43.3)	15 (37.5)
No	304 (54.5)	36 (45.0)	40 (50.0)	203 (56.7)	25 (62.5)
Husband employed (,*n = 441*)					
Employed	415 (94.1)	66 (95.6)	64 (98.5)	262 (94.2)	23 (79.3)
Un-employed	26 (5.9)	3 (4.4)	1 (1.5)	16 (5.8)	6 (20.7)
Parity					
I or II	217 (39.0)	37 (46.3)	41 (51.2)	124 (34.6)	15 (37.5)
≥ III	341 (61.0)	43 (53.7)	39 (48.8)	234 (65.4)	25 (62.5)
Disclosed HIV sero-status					
Yes	376 (67.4)	44 (55.0)	59 (73.7)	259 (72.4)	14 (35.0)
No	182 (32.6)	36 (45.0)	21 (26.3)	99 (27.6)	26 (65.0)
Disclosed HIV sero-status to spouse (among married/co-habiting, *n* = 441)					
Yes	194 (44.0)	34 (49.3)	33 (50.8)	126 (45.3)	1 (3.5)
No	247 (56.0)	35 (50.7)	32 (49.2)	152 (54.7)	28 (96.5)
Attending any health clinic/organization giving HIV related services					
Yes	435 (78.0)	50 (62.5)	67 (83.7)	303 (84.6)	15 (37.5)
No	123 (22.0)	30 (37.5)	13 (16.3)	55 (15.4)	25 (62.5)

**Fig. 2:**
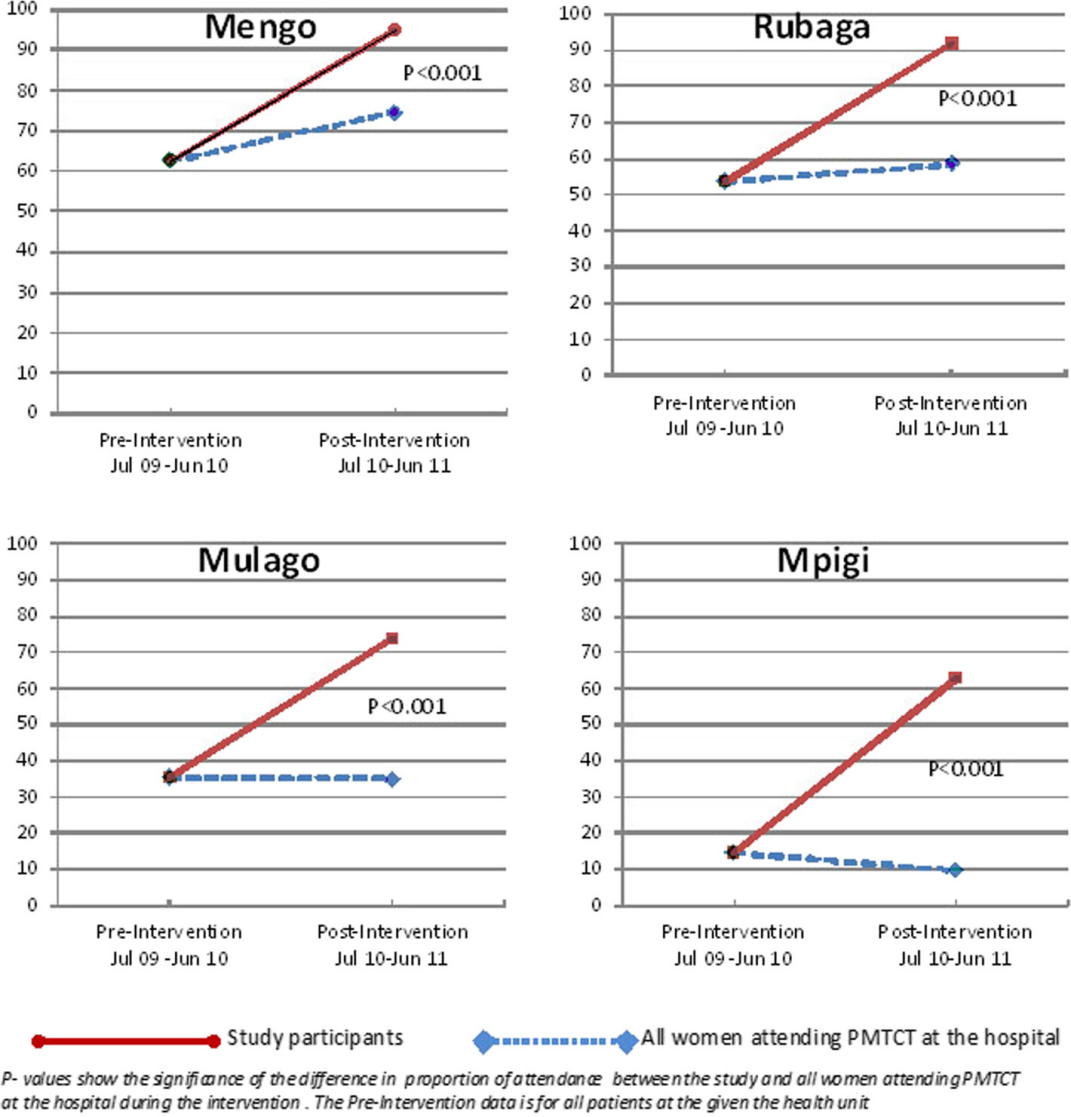
Six-weeks Postnatal visit among women attending PMTCT at the four study site Health units

After implementation of the intervention for one year, there was a significant increase in the proportion of women who returned to the 6-week postnatal visit for study participants compared to the whole clinic population in all the four study sites, from the baseline of 37.7 % to 78.5 % and 39.1 % for study participants and whole clinic population respectively, an incremental difference of 39.4 % (*P* < 0.001). At Mengo, the proportion attending 6-week PNC increased from the baseline of 62.5 % to 94.7 % and 74.7 % for study participants and whole clinic population respectively (incremental difference of 20.0 %:*P* < 0.001). At Rubaga, 6-week PNC attendance increased from the baseline of 53.7 % to 91.9 % and 58.3 % for study participants and whole clinic population respectively (incremental difference of 33.6 %: *P* < 0.001). At Mpigi, six-week PNC attendance reduced from the baseline of 14.6 % to 9.8 % in the whole clinic population but increased to 62.9 % for study participants (incremental difference of 53.1 %: *P* < 0.001) and at Mulago there was no observed change in PNC attendance in the whole clinic population from baseline of 35.1 % to 35.4 % but significant increase among study participants to 73.4 % (incremental difference of 38.0 %: *P* < 0.001) (Fig. 2).

There was no difference in PNC attendance with increase in age (adjusted OR 1.02: *p* = 0.277), and no significant difference in PNC attendance between educated and un-educated women (adjusted OR 1.39: *p* = 0.146). Six week PNC attendance was not statistically different among un-employed and employed women (adjusted OR 0.72: *p* = 0.16), but un-employment of a husband was associated with lower PNC attendance compared to employed husbands (un-adjusted OR 0.32: *p* = 0.008). Six week PNC attendance did not differ significantly by HIV status disclosure (unadjusted OR 0.79: *p* = 0.29) and not different among women attending or receiving HIV services or care at the time of enrolment in the study (unadjusted OR 0.75: *p* = 0.26). Women from urban Kampala were more likely to return for their 6-week visit compared to women from rural Mpigi (adjusted OR 2.13: *p* = 0.041). (Table 2).

**Table 2:** Factors associated with 6 weeks PNC attendance among women enrolled in the study (*N* = 511^a^)

	Attended PNC	Unadjusted		Adjusted	
	*n* (%)	OR (95%CI)	*P* value	OR (95%CI)	*P* value
a) Demographics and baseline factors					
Age (yrs), mean(SD)	27 (5.1)	1.03 (0.98-1.07)	0.211	1.02 (0.98-1.07)	0.277
Marital status					
Married/cohabiting	410 (78.3)	1			
Single/separated/widowed	101 (79.2)	1.05 (0.61-1.80)	0.841		
Education					
None/Primary	250 (75.6)	1		1	
Secondary/Post-secondary	261 (81.2)	1.40 (0.91-2.13)	0.123	1.39 (0.89-2.16)	0.146
Occupation					
Employed	236 (82.2)	1		1	
Un-employed	275(75.3)	0.66 (0.43-1.01)	0.058	0.72 (0.46-1.14)	0.160
Occupation of husband (for married/cohabiting women*, n = 410*)					
Employed	385 (79.7)	1			
Un-employed	25 (56.0)	0.32 (0.14-0.74)	0.008		
Parity					
One,two	195 (77.4)	1			
≥ three	316 (79.1)	1.10 (0.72-1.70)	0.654		
Disclosed HIV sero-status					
Yes	342 (79.8)	1			
no	169 (75.7)	0.79 (0.51-1.22)	0.291		
Attending any health clinic/organization giving HIV related services as at enrollment					
Yes	401 (79.6)	1			
No	110 (74.6)	0.75 (0.46-1.23)	0.259		
d) Setting					
Rural (Mpigi)	35 (62.9)	1		1	
Urban (Rubaga, Mengo & Mulago)	476 (79.6)	2.31 (1.12-4.74)	0.023	2.13 (1.03-4.43)	0.041

^a^For 6-weeks PNC visit we considered women who registered live birth, minus infant or maternal death prior to 6 weeks (*N* = 511)

Only women who attended PNC in the 6 weeks window (5-8weeks) were considered to have attended 6 week PNC visit (401)

In the multivariate analyses we adjusted for age and other variables with *P* < 0.2 in bivariate analyses

Overall across all study sited, the proportion of HIV exposed infants who had an HIV DNA PCR test by 14 weeks of age increased significantly during the study period from the baseline of 53.6 % to 86.3 % and 65.8 % for study participants and whole clinic population respectively, a statistically significant incremental difference of 20.5 % (*p* < 0.001). Significantly higher infant HIV testing was registered among study participants compared to the whole clinic population at: Rubaga hospital (95.9 % vs 80.9 % respectively: *p* = 0.001) and Mulago hospital (82.3 % vs 54.1 % respectively: *p*-value < 0. 001) during the study period. However, infant HIV testing was lower among study participants than the whole clinic population at Mengo hospital (97.3 % vs 99.3 % respectively: *p* = 0.106) and Mpigi health center (80.0 % vs 95.4 % and: *p* = 0.007) during the study period. Infant HIV infection rate among study participants was similar among the four study sites, with 13/511 (2.5 %) of all infants tested found to be HIV infected. (Table 3).

**Table 3:** HIV DNA PCR testing for infants up to 14 weeks of age from July 2010 to June 2011

	All HIV-exposed infants at health unit^b^		Study participants		
	Infants HIV tested/ live birth (%)	Infants HIV tested / live birth, *n *(%)	Infants HIV tested/ live birth^a^ *n *(%)	HIV positive infants, *n *(%)	Proportional difference in infant HIV testing between study participants and All exposed infants at the hospital
Site	(Baseline)	(Post intervention)	(Post intervention)		% (*P*-value)
Mengo	304/374 (81.2)	439/442 (99.3)	73/75 (97.3)	2/75 (2.7)	-2.0 (*P* = 0.106)
Rubaga	374/506 (73.9)	437/540 (80.9)	71/74 (95.9)	2/74 (2.7)	15.0 (*P* = 0.001)
Mulago	1055/2411 (43.7)	1179/2178 (54.1)	269/327 (82.3)	8/327 (2.4)	28.2 (*P* < 0.001)
Mpigi	81/96 (84.4)	83/87 (95.4)	28/35 (80.0)	1 /35 (2.9)	-15.4 (*P* = 0.007)
Total	1814/3387 (53.6)	2138/3247 (65.8)	441/511 (86.3)	13 /511 (2.5)	20.5 (*P* < 0.001)

^a^For study participants we considered all registered live birth (whether at study site health unit or anywhere else), less infant or maternal death prior to 6 weeks (*N*=511)

^b^For all HIV-exposed infants at the site health units, live births records were only registered for women delivering at the unit (Information for infant death before 6-weeks was not available)

## Discussion

These data show that use of peers, influential community lay persons and VHT members was effective in increasing 6-week postnatal attendance from 37.1 % at baseline to 78.5 % among study participants compared to only 39.1 % among the whole clinic population. This combined intervention also significantly increased early infant diagnosis from 53.6 % at baseline to 86.3 % among study participants compared to only 65.8 % for the whole clinic population among registered live births. We believe this observation was a result of peers mothers support, as HIV positive mothers felt more comfortable disclosing to the peers than the community lay persons. Some participants did not disclose their HIV status beyond the hospital and study staff and declined to be followed by the community lay women. This led to the use of more peer home visits compared with the lay persons. Therefore, the lay person spent more time educating and sensitizing the communities. We believe this resulted in the observed increase in postnatal follow up even in the general population. In addition there were other community activities that targeted the general population and hence influenced the improvement among the whole clinic population, albeit marginal.

These findings are in agreement with previous studies and program data which have shown that use of peer-mothers/educators and community lay persons can significantly improve knowledge and retention in long-term HIV care among women living with HIV [19, 20]. In this study, it is possible that participants identified more closely with the peers as they were HIV infected and better equipped to handle HIV related issues including stigma and discrimination, than the community lay persons or VHT members. This level of task shifting/sharing is effective in expanding the work force to support referral and linkage to other services within and outside the health unit in the community. VHT members and community lay persons were facilitated with transport to carry out the specific study related activities but no additional allowances were provided. Peers on the other hand, because they spent up to 8 hours at the sites, were given a small monthly stipend to cater for daily transport and lunch. This additional small expense for these very useful volunteers may be sustained by the health unit through re-allocation from the community out outreach allowances or through partnerships with community based organizations, hence freeing the health workers to concentrate on health services at the health unit. The additional fees required to support the peers is a worthwhile investment if postnatal follow up of HIV infected mothers and their infants is improved with increased EID.

The different health centers selected for the study demonstrate how the mother’s social status, accessing services at urban, rural and private health facilities may influence postnatal attendance. Mengo and Rubaga are urban, private hospitals and clients attending these units were more educated and more likely to be employed and both had a PNC return of over 50 % at baseline and over 90 % among study participants after the intervention. Rural Mpigi health center had younger mothers, less educated, unemployed and a very low baseline postnatal follow up of 14.6 %. Mulago Hospital, though urban, is a public referral hospital and receives women who are less educated and with lower social economic status, which may explain the lower PNC attendance both at baseline and after the study intervention. However, there was still a significant increase in postnatal attendance among study participants at all sites after implementation of intervention. On the other hand, Mpigi, the rural health unit appeared to benefit most from the addition of peer support and stronger community linkage as seen by the absolute increase in attendance of 53.1 % from baseline. The extra support may have added valuable knowledge, interpretation of medical information and appointments to clients in rural areas or the less educated.

Infant HIV testing increased in all the four clinics for both the whole clinic population and study participants, a result attributed to the robust National EID program that began in 2008 to support referral, linkage, HIV testing and return for HIV results following testing [23]. At the Mulago public hospital, this success was less than the other two urban clinics, possibly because most deliveries at the hospital are referrals from other clinics, further underscoring the importance of inter-clinic linkages for clear documentation and reporting. At rural Mpigi health center, we observed a significant reduction in HIV testing between study participants and the whole clinic population, a finding we believe may be due to small numbers and very low clinic return in the whole Mpigi PMTCT clinic population, hence providing higher spill over influence from study intervention.

Still birth and infant death were reported in 6.6 % (37/558) of the study participants, a figure that is slightly higher than the national infant and child mortality [24] with most infant deaths occurring in the first one week of life. This observation we believe may be due to the rigorous tracking done by the study peers to report all the death and may not be achieved from routine national reporting. Loss to follow up or missed visit at 6 weeks was 21.5 %, a figure that is consistent with that reported in other studies [25]. A few, 2.7 % of the participants opted to take their children to other health centers where comprehensive services were offered (documented review by peers during home visit), a finding that calls for inter-clinic linkage through either an electronic central database or one-on-one clinic update whenever a client attends another clinic. We believe without active participant tracking and community follow up there may be higher documented rates of loss to follow up due to lack of information to accurately document infant death, maternal death or attendance in other health units.

The study had some limitations. The reference data collected from the clinic registers was incomplete for a few entries, especially the PNC registers and this may lead to over-estimation of the findings. We also did not disaggregate study participants’ data from the whole clinic population report, so the proportion from the rest of the clinic includes data for study participants. The fact that we still observed a difference under this consideration indicates a possibility of an even greater effect of the intervention. We considered different clinics with different background characteristics but these may not be generalizable to areas with different characteristics or health seeking behavior. Our intervention was primarily health unit peer focused since some mothers declined to disclose their HIV status to the community lay persons and hence were not visited by the lay persons. This resulted in having more home visits by peer and leaving the lay persons to concentrate on general health education and sensitization.

## Conclusion

Peers and community lay persons are an invaluable addition to the traditional health care team in low resource settings where the health care work force may not be adequate. Their role in health education, linkage and referral improves early postnatal attendance and EID, which in turn leads to better service delivery and care for HIV infected women and their infants.

## Abbreviations

PMTCT: Prevention of Mother to Child Health Organization
EID: Early Infant Diagnosis of HIV
VHT: Village Health Team
DNA: Deoxyribonucleic acid
PCR: Polymerase Chain Reaction
3TC: Lamivudine
AIDS: Acquired Immunodeficiency syndrome
ANC: Antenatal Care
ART: Anti-retroviral therapy
AZT: Zidovudine
CBO: Community based Organization
HIV: Human Immunodeficiency Virus
IQR: Inter-quartile range
ITN: Insecticide Treated net
MoH: Uganda Ministry of health
NVP: Nevirapine
PNC: Postnatal Care
RH: Reproductive health
sdNVP: Single-dose Nevirapine
WHO: World health Organization
